# Editorial: Plant diversity patterns and drivers

**DOI:** 10.3389/fpls.2024.1474649

**Published:** 2024-08-22

**Authors:** Le Ma, Liwen Shao, Hailong Bao, Qing Zhang

**Affiliations:** ^1^ Inner Mongolia University, Hohhot, China; ^2^ Inner Mongolia Pratacultural Technology Innovation Center Co. Ltd, Hohhot, China

**Keywords:** plant community, driving mechanisms, population dynamics, distribution pattern, ecosystem function

Global biodiversity is currently experiencing an unprecedented decline, primarily due to the impacts of climate change and human activities (Kannan and James, 2009; Ramirez et al., 2017; Su et al., 2021). The sharp decline in biodiversity not only threatens the health and stability of ecosystems but also severely impacts the ecosystem services that human societies depend on (Brockerhoff et al., 2017; Lovejoy, 2008; Pereira et al., 2024). Therefore, assessing historical changes and predicting future trends in biodiversity are crucial for developing effective conservation strategies (Dunlop, 2013; Harrison, 2010). Plant communities, central to ecosystem structure and essential for material cycles, are vital providers of ecosystem functions and services (Dietrich et al., 2024; Jepson and Canney, 2001). The richness and stability of plant communities are crucial for maintaining ecological balance and enhancing ecological processes (Kardol et al., 2010; Minden et al., 2016; Willig, 2011). This topic includes 34 articles that explore the distribution patterns of plant diversity and identify the key drivers behind these changes. These findings not only offer new insights into the study of plant diversity but also underscore the main challenges faced by current research, establishing a solid foundation for future research directions, strategy development, and conservation efforts (Grime, 2006; Suding et al., 2008).

This Research Topic explores various aspects of plant diversity. The studies by Umair et al. focused on traditional classifications of species richness and abundance, while research by Chen et al. and Shrestha et al. concentrated on functional diversity, revealing the characteristics of ecosystem functions and services. Studies by Wei et al. and Sha et al. discussed phylogenetic diversity, providing insights into the historical processes of species formation and expansion, as well as the evolutionary potential to adapt to environmental changes. Multidimensional studies of plant diversity enhance our understanding of the complexity of ecosystems and offer interdisciplinary perspectives for ecology, evolutionary biology, and conservation biology. These insights are crucial for protecting plant diversity and maintaining ecosystem functions (Burdon et al., 2006; Soliveres et al., 2015).

The articles in this Research Topic utilize a range of ecological research methods for data collection, including: (1) Long-term field monitoring by establishing fixed plots for various activities such as plot surveys and phenological observations. For example, Huang et al. established fixed forest dynamics monitoring plots to collect and record data on plants, climate, and soil factors. Additionally, Zhang et al. and Wu et al. quantified the specific impacts of grazing on species richness through grazing gradient experiments. (2) Controlled experiments and microcosm experiments conducted indoors allow for the study of plant responses to environmental changes under controlled conditions. For example, Zhu et al. analyzed the impact of population genetic diversity on gas fluxes in soil systems through a microcosm experiment with water celery genotypes. (3) Model development and analysis, including ecological niche models and geographically weighted regression models, help us understand the relationships between plant richness and climatic factors. For instance, Sun et al. used geographically weighted regression models to analyze these relationships, while Zhou et al. predicted the spatial and temporal distributions of species using the Maxent model. (4) Remote sensing technologies, satellite imagery analysis, and drone monitoring, which provide macroscopic observation and analysis methods for plant diversity, like Zhang et al. used satellite images to study urban green spaces. (5) Molecular biology techniques, such as DNA sequencing and transcriptome analysis, reveal the genetic structure and evolutionary history of plant species. For instance, Yu et al. and Yan et al. sequenced DNA fragments of plants to analyze genetic diversity, while Sha et al. elucidated species’ evolutionary processes through transcriptome analysis. These methods can be used independently or in combination, providing a comprehensive and in-depth perspective on the complexity and dynamic changes in plant diversity.

This Research Topic focuses on exploring the driving factors behind plant diversity. Firstly, climate change is identified as a major driver shaping plant diversity (Harrison et al., 2020). Several articles discuss the impact of contemporary climate indicators, such as drought and precipitation, on plant diversity, species growth, and distribution. Historical climate has also been identified as a key factor affecting plant diversity, as found by Yu et al. in the population dynamics of two typical desert shrubs, white thorn and bubble thorn, in Northern China were influenced by Quaternary glacial climate oscillations. Similarly, Zhang et al. ‘s study on the distribution of C4 species in China highlights the role of historical climate in shaping species distribution and diversity. Secondly, soil factors are extensively studied as a driving mechanism. Gong et al. analyzed nutrient changes in the soil during the restoration of the Maowusu sandy land in Inner Mongolia, revealing that soil carbon and nitrogen promote plant diversity, while phosphorus acts as a limiting factor. Wei et al. found that soil physicochemical properties significantly influence the variation in soil algal communities. Additionally, plant diversity is often driven by a combination of climate and soil factors. For instance, Liu et al. discovered that soil and plants jointly influence the secondary succession process of shrub-herb communities. Similarly, Huang et al. found that the functional traits of 215 woody plants across four climatic zones in China are significantly affected by both climate and soil factors. Thirdly, human activities and landscape changes significantly impact plant diversity. For instance, Zhang et al., conducted grazing intensity experiments, highlighting the effects of grazing on plant community diversity and productivity. Bellini et al., through paleobotanical exploration, discussed the relationship between ancient agricultural practices and plant population dynamics, illustrating how plants preadapted to human activities. Zhong et al. found that island size significantly influences the dynamics of woody plant seedlings in Thousand Island Lake, particularly affecting seedling mortality. Shrestha et al. revealed that habitat fragmentation alters the coloration of angiosperm flowers, thereby affecting pollinators’ ability to recognize them. Therefore, understanding how human activities and landscape factors drive plant diversity is crucial for developing land planning and ecological conservation strategies.

In summary, this research focuses on exploring the patterns and drivers of plant diversity. As a frontier in plant science research, future studies are recommended to strengthen in the following five areas: First, plants respond to changing environments in various ways, and their adaptations are often a result of long historical processes, thus requiring support from long-term experiments and monitoring. Second, although diversity in species, function, and phylogeny has been explored, current research is often isolated to one aspect; future studies should focus on the relationship between these three dimensions of diversity and their coupled elucidation of plant diversity (Borer et al., 2012; Hu et al., 2023). Third, as plant diversity distribution patterns are driven by different mechanisms, further clarification of the links between different mechanisms is recommended to more comprehensively reveal the mechanisms sustaining biodiversity (Jiang et al., 2022). Fourth, it is advisable to strengthen applied research, combining theory and practice, and, based on elucidating plant diversity patterns and driving mechanisms, to carry out conservation applications for plant diversity (Barthlott et al., 2007; Viers et al., 2012). Lastly, although this research theme focuses on terrestrial ecosystems, future studies should broaden to more extensive ecosystems, especially aquatic and marine ecosystems, to more comprehensively understand the patterns and drivers of plant diversity.
